# Metabolic alterations in patients with essential tremor before and after deep brain stimulation: keys to understanding tremor using magnetic resonance spectroscopy

**DOI:** 10.3389/fneur.2025.1544688

**Published:** 2025-07-31

**Authors:** Nathanael Göransson, Sofie Tapper, Peter Lundberg, Peter Zsigmond, Anders Tisell

**Affiliations:** ^1^Department of Biomedical Engineering, Linköping University, Linköping, Sweden; ^2^Department of Biomedical and Clinical Sciences, Linköping University, Linköping, Sweden; ^3^Department of Medical Radiation Physics, Linköping University, Linköping, Sweden; ^4^Department of Health, Medicine and Caring Sciences, Linköping University, Linköping, Sweden; ^5^Center for Medical Image Science and Visualization (CMIV), Linköping University, Linköping, Sweden; ^6^Department of Radiology, Linköping University, Linköping, Sweden

**Keywords:** deep brain stimulation, essential tremor, magnetic resonance spectroscopy, functional neurosurgery, movement disorder surgery

## Abstract

**Introduction:**

The pathophysiology behind essential tremor (ET) and the mechanisms behind the clinical effect after deep brain stimulation (DBS) is not fully understood. This article aims to increase the understanding of ET pathophysiology and the mechanisms behind DBS using magnetic resonance spectroscopy (^1^H-MRS). Patients with ET underwent MRS scans of the cerebellum and thalamus before and after DBS, and the results were compared to a healthy control group.

**Methods:**

Ten ET patients and seven healthy controls were included. Preoperatively and ~5 months postoperatively, single-voxel MRS was performed on a 1.5 T (tesla) system. Voxels were placed in the thalamus (14 × 13 × 13 mm^3^), dentate nucleus (13 × 13 × 13 mm^3^), and cerebellar cortex (13 × 13 × 13 mm^3^). Metabolite concentrations of total N-acetylaspartate + N-acetyl-aspartyl-glutamate (tNA), total creatine + phosphocreatine (tCr), total choline + phosphocholine + glycerophosphocholine (tCho), and total glutamate and glutamine, which together constitute Glx, were quantified. The patients were evaluated with the *Essential Tremor Rating Scale* (ETRS) preoperatively and postoperatively.

**Results:**

A total of 14 leads were implanted, and ETRS scores improved significantly following surgery. Thalamic tNA concentrations reduced significantly within the patient group after surgery, as well as in comparison to healthy control values. A significant difference in tNA concentration following surgery was observed only in the thalamus that had been targeted with a lead, not elsewhere. No additional differences in metabolite concentrations (tCr, tCho, Glx) were observed in the thalamic voxel, and none of the studied metabolites (tNA, tCr, tCho, Glx) showed detectable differences in the cerebellar voxels (dentate nucleus and cerebellar cortex).

**Conclusion:**

In a highly selected patient group affected by ET, we present novel metabolite information using MRS. Specifically, a reduced thalamic tNA concentration was observed on the lead-implanted side following DBS, suggesting a possible treatment effect.

## Introduction

Essential tremor (ET) is one of the most common movement disorders (1) characterized by postural and action tremor (2). The tremor can be progressive (3), and deep brain stimulation (DBS) is used to reduce symptoms in the subgroup of patients with the most disabling tremor (4). The pathophysiology behind ET is not fully understood (5), and the mechanism behind the remarkable clinical effect after DBS is debated (6).

For many years, ET was viewed as a benign monosymptomatic disorder. However, a growing body of evidence indicates that ET is not a monosymptomatic disorder, but rather a progressive condition that is clinically heterogeneous (7). Neuroimaging studies suggest that the thalamus and cerebellum are involved in the pathogenesis of ET (5) and the cerebello-thalamo-cortical network is targeted with DBS by placing the lead in the ventral intermediate nucleus (Vim) (4).

Proton magnetic resonance spectroscopy (^1^H-MRS) is a non-invasive method that characterizes the neurometabolic disposition of the brain *in vivo* (8, 9). It can be used in the diagnosis of neurological conditions and to support clinical decision-making (10). By using MRS, the metabolite concentration of total creatine and phosphocreatine (tCr), total choline, phosphocholine, and glycerophosphocholine (tCho), N-acetylaspartate and N-acetyl-aspartyl-glutamate (tNA), as well as glutamate (Glu) and glutamine (Gln), which together are referred to as (Glx), can be measured. These measurable metabolites can provide markers associated with different clinical conditions. tCr is involved in cell energy homeostasis, and tCho is a marker for membrane turnover and changes in cell density (11). tNA is mainly a neuron-specific metabolite (12), and Glx contains Glu, which is the most abundant excitatory neurotransmitter (11).

Patients with ET have been found to have decreased ratios of tNA/tCr in the cerebellum in previous studies (13, 14), and an asymmetry in thalamic tNA/tCr was found in an ET group with a lower ratio in the thalamus, coupled to the arm most affected by tremor (15). These findings all suggest neurodegenerative changes. Furthermore, there are also evidence of functional changes. Increased thalamic Glx levels have been described in ET patients (16), and both decreased cerebellar cortical tNA/tCr ratio (14) and increased thalamic Glx level were correlated with tremor severity (16). Furthermore, the cerebellar GABA+/Glx ratio was positively correlated with tremor severity (8).

Studying metabolites in specific areas of the brain that appear to be involved in the pathophysiology before and after surgery can contribute to understanding ET and DBS. Patients planned for DBS surgery usually have a more severe form of ET compared to patients included in previous studies (13–16). Additionally, metabolite levels in this patient group, measured with MRS pre- and postoperatively compared to DBS, have not, to the best of our knowledge, been previously investigated.

This prospective pilot study investigated ET patients with MRS in the cerebellum and thalamus, before and after DBS surgery, comparing them to a healthy control group. The study also evaluates whether the severity of tremor correlates with the examined metabolites. Thus, this article aims to increase the understanding of ET pathophysiology and the mechanisms behind DBS.

## Materials and methods

### Patients

All patients who were referred and eligible for surgery were included for ~1 year. They were all diagnosed and referred to the neurosurgical department by a movement disorder specialist. In total, 10 ET patients planned for DBS surgery were included in this prospective pilot study, with a median age of 61.1 years (range: 41.0–75.9), seven of whom were male. All participants experienced severe tremors in the upper extremities and had not achieved adequate symptom relief with pharmacological treatment. Tremor-reducing medication was discontinued 1 week prior to surgery in accordance with hospital policies, and the MRS examination was performed the day before surgery. Given that ~1 week had passed since discontinuation, it is, therefore, expected that the patients' medications were fully metabolized when the MRS examination was conducted. The control group consisted of seven healthy controls (HCs), with a median age of 66.2 years and a range of 41.6–76.7, and six of them were male. All subjects were Caucasian and right-handed.

Tremor severity was evaluated according to a sub-score of the Fahn, Tolosa, and Marin essential tremor rating scale (ETRS) (17) representing upper extremity tremor and hand function, the dominant extremity (part A item 5 and part B items 10–14). The total sum was calculated with a maximum value of 32 points. A greater score suggests more severe symptoms. The patient group has previously been described in a previous scientific report (8).

### Ethics

The local ethics committee at the University Hospital in Linköping (Ref. No. 2013/ 403–31, P. Zsigmond) approved the study. All subjects (patients and healthy controls) gave their written consent after receiving information about the study.

### Surgery

Surgery was performed using indirect targeting in SurgiPlan (Elekta Instruments AB, Stockholm, Sweden). Targeting was planned on the T2-weighted (T2w) magnetic resonance images (MRIs). Indirect targeting of the VIM was performed using standardized coordinates for the left and right thalamic side, based on the Schaltenbrand–Wahren human brain atlas. The DBS equipment used was from Medtronic (Minneapolis, MN, USA), using electrode lead 3389 with the extension kit 37086. The implantable pulse generator Activa PC 37601 is for bilateral leads, and Activa SC 37603 is for unilateral leads. The surgical procedure performed is described in a previous study (18).

### Magnetic resonance measurements

Preoperatively and also ~5 months postoperatively, MRI examinations were performed on a 1.5 T Achieva dStream MR scanner (Philips Healthcare, Best, The Netherlands), using a Transmit/Receive head coil (as stated in the MR conditions for the implant). The DBS system was switched off just prior to the postoperative MRS, which was subsequently performed within minutes. The patients were instructed to remain still during the MRS examination and refrain from any voluntary movements.

Single-voxel MRS was performed using point-resolved spectroscopy (PRESS), an acquisition technique for localization and creating a volume of interest (VOI). The sequence settings were as follows: echo time (t_TE_) of 25 ms, repetition time (t_TR_) of 3 s, 1 kHz sampling frequency, and 128 water-suppressed transients were averaged. Water suppression was achieved using the excitation method, and eight non-water-suppressed transients were used as the reference signal. The VOIs were planned using a T2-weighted (T2w) turbo spin echo sequence (TSE), and the voxel was positioned for the tNA-resonance chemical shift. Axial; TSE factor 15, reconstructed resolution 0.65^*^0.65^*^2 mm^3^, field of view (FOV) 250^*^190^*^168 mm^3^, t_TE_ 80 ms, t_TR_ 8 s. Sagittal; TSE factor 23, reconstructed resolution 0.9^*^0.9^*^2 mm^3^, FOV 230^*^230^*^66 mm^3^, t_TE_ 110 ms, t_TR_ 8 s. Coronal; TSE factor 23, reconstructed resolution 0.9^*^0.9^*^3 mm^3^, FOV 230^*^230^*^120 mm^3^, t_TE_ 110 ms, t_TR_ 8.8 s. An experienced MR operator assessed the quality of the spectra.

**Figure 1:**
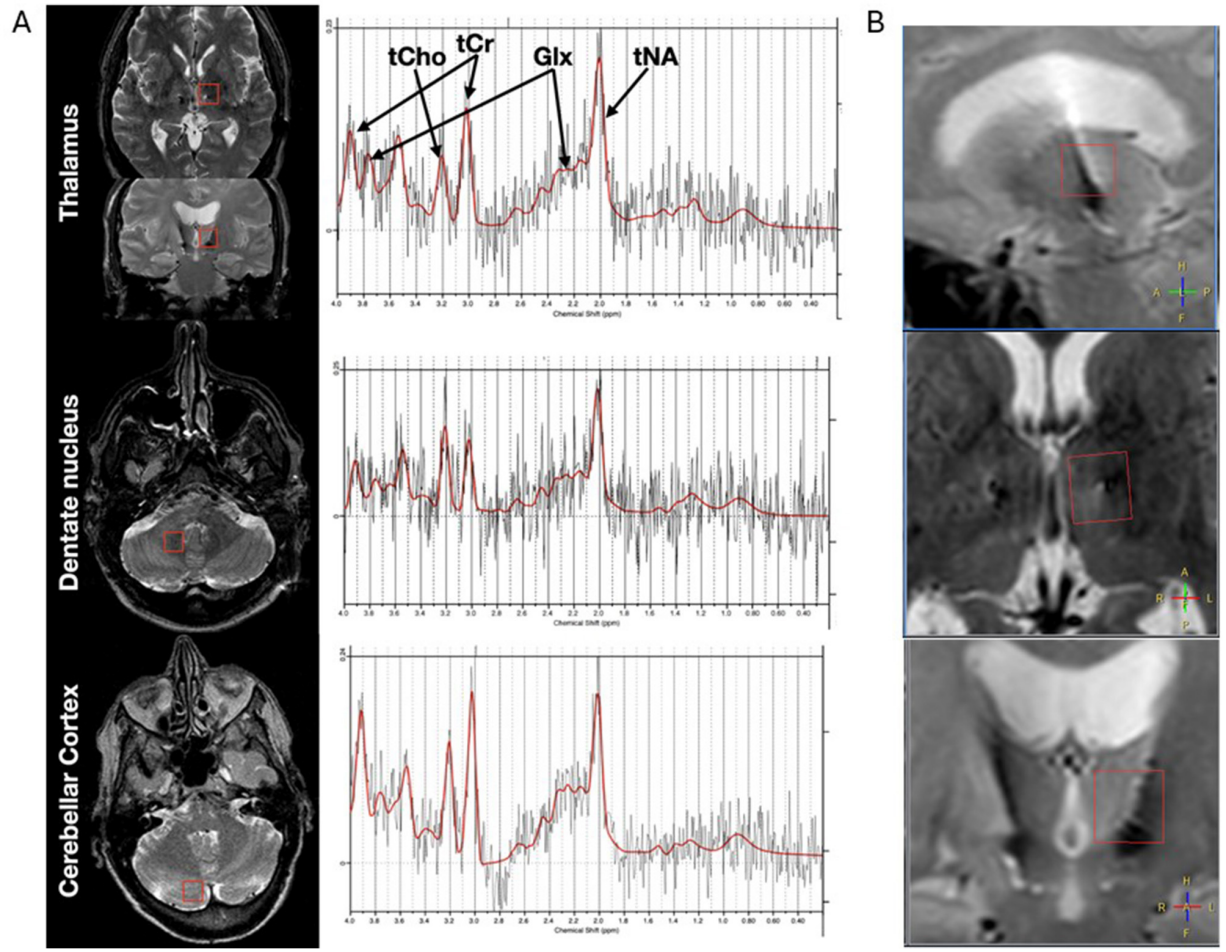
**(A)** Illustrates voxel placement and typical spectrum for the left thalamus, the right dentate nucleus, and the right cerebellar cortex. tCho (phosphocholine and glycerophosphocholine), tCr (creatine and phosphocreatine), Glx (glutamate and glutamine), and tNA (N-acetylaspartate and N-acetyl-aspartyl-glutamate). The voxel placement, exemplified in **(A)**, was chosen to reflect the pathway of the cerebello-thalamic tract, which decussates to the contralateral thalamus. The voxels were positioned symmetrically in the corresponding anatomical regions on the other side. **(B)** Demonstrates that the lead is visible as a susceptibility artifact in all directions within the thalamus voxel, as shown in the sagittal, axial, and coronal views.

A total of six voxels per examination were placed bilaterally in the thalamus (14 × 13 × 13 mm^3^), dentate nucleus (13 × 13 × 13 mm^3^), and cerebellar cortex (13 × 13 × 13 mm^3^). The voxels were placed by two trained neurosurgeons (NG and PZ) to enable a comparable and reproducible bilateral voxel location in the anatomy, and are shown in Figure 1.

The thalamus voxel was placed in the superior/medial/anterior part of the thalamus. This voxel placement focuses on the motor part of the thalamus, excluding cerebral spinal fluid, and minimizes the contribution of other adjacent anatomical structures. The lead was visually observed from the susceptibility artifact, as shown in Figure 1. The DBS system was switched off just prior to the postoperative MRS.

Metabolite concentrations of tNA (N-acetylaspartate + N-acetyl-aspartyl-glutamate), tCr (creatine + phosphocreatine), tCho (choline, phosphocholine, and glycerophosphocholine), and Glx (Glu+Gln) were quantified using LCModel (Version 6.3-1L). We used the unsuppressed water spectrum as an internal reference instead of using tCr, since tCr cannot be assumed to be stable (11). When comparing concentrations using a ratio, it can be challenging to determine which concentration is responsible for the observed difference (9). The potential influence of edema was investigated in both the preoperative and postoperative settings using T2w images. No patients presented with edema either preoperatively or 5 months postoperatively.

### Statistical analysis

Metabolites were assessed for normal distribution using the Shapiro–Wilks test. Mann–Whitney *U*-tests were used to compare the non-parametric data, and *t*-tests were used to compare normally distributed data. In the patient group, Glx levels in the thalamus preoperatively and tCho levels in the cerebellar cortex postoperatively were not normally distributed. These metabolites were therefore analyzed using the Mann–Whitney *U*-test, while the remaining metabolites were assessed using parametric statistical methods. Furthermore, metabolite concentration was assessed for lateral differences. No lateral metabolite asymmetry was observed preoperatively in the patient group in the thalamus, dentate nucleus, or cerebellar cortex. Therefore, the mean concentration of bilateral voxels was used.

Preoperative concentrations of metabolites in patients were compared to those of the healthy control group, followed by an examination of postoperative concentrations after DBS. Differences at the group level between patients and healthy controls were examined using the *t*-test or the Mann–Whitney *U*-test. A separate test was performed for each metabolite concentration (tCr, tNA, tCho, Glx) in each voxel (thalamus, dentate nucleus, and cerebellar cortex). The tests were paired when evaluating pre- and postoperative metabolite concentrations in patients; otherwise, they were unpaired. The severity of the movement disorder was analyzed before and after DBS surgery, and its relationship to metabolite concentrations was assessed using Spearman's correlation coefficient, including only those metabolites that showed significant differences in the analyses described above. We consider a p-value of < 0.05 to be significant for all tests.

## Results

In total, 14 leads were implanted in nine patients. One patient declined surgery and was therefore excluded from the postoperative analysis. The target used was Vim, and five received bilateral implantation.

Preoperatively and postoperatively [median 5.2 months after surgery, range (3.4–6.8)], an MRS examination was performed with the placement of voxels for metabolite calculation.

The median ETRS was preoperatively 17 (range 14–23), and the postoperative corresponding value was 4 (range 0–8). The median ETRS difference (postoperative – preoperative ETRS) was a reduction of 14 points (range 8–17), *p* = 0.004 (Mann–Whitney *U*-test).

### MRS examination

Two left and one right dentate nucleus voxels were excluded from the healthy control group due to poor spectral quality and a disproportionately posterior position of a voxel compared to the rest. All other healthy and preoperative spectra passed the visual inspection, and corresponding computed concentrations were used in further analyses.

Postoperatively, two right and two left dentate nucleus voxels and one right and two left voxels of the thalamus were discarded after quality inspection of the fitted spectra. Additionally, one patient was missing data in the right cerebellar cortex. The low quality of some spectra was most likely due to patient movement in the scanner. The quality of the spectra was consistent regardless of whether the electrode was located within the voxel or not. Typical voxel placement and spectrum for the thalamus, dentate nucleus, and cerebellar cortex are shown in Figure 1.

### Metabolite concentration changes

**Figure 2:**
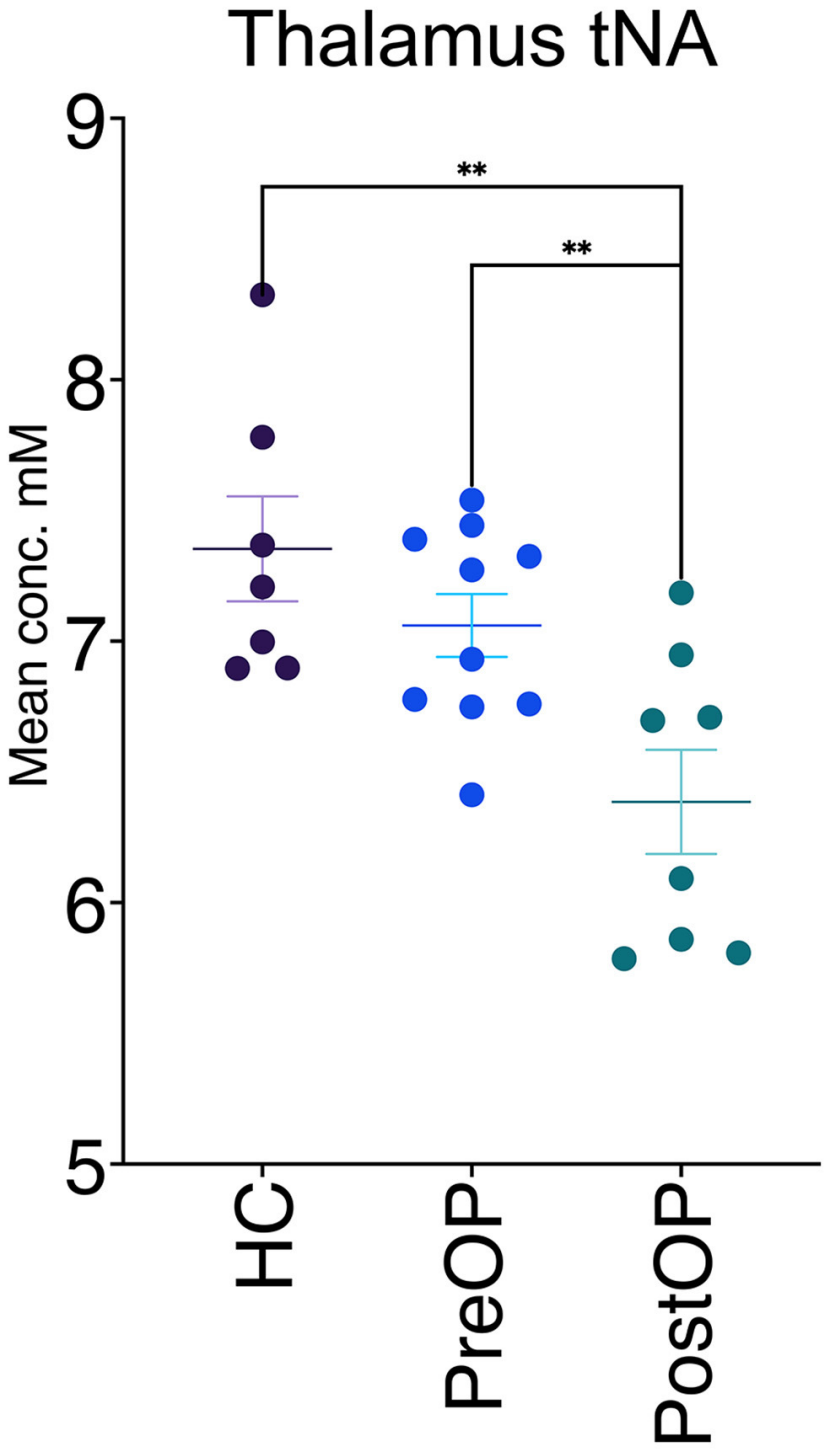
Metabolite concentration in the thalamus (tNA; N-acetylaspartate and N-acetyl-aspartyl glutamate). A significant change in tNA was observed post-DBS, both in comparison to pre-DBS values and to healthy controls (one patient declined surgery, and bilateral measurements were excluded for one patient in the thalamus postoperatively).

**Figure 3:**
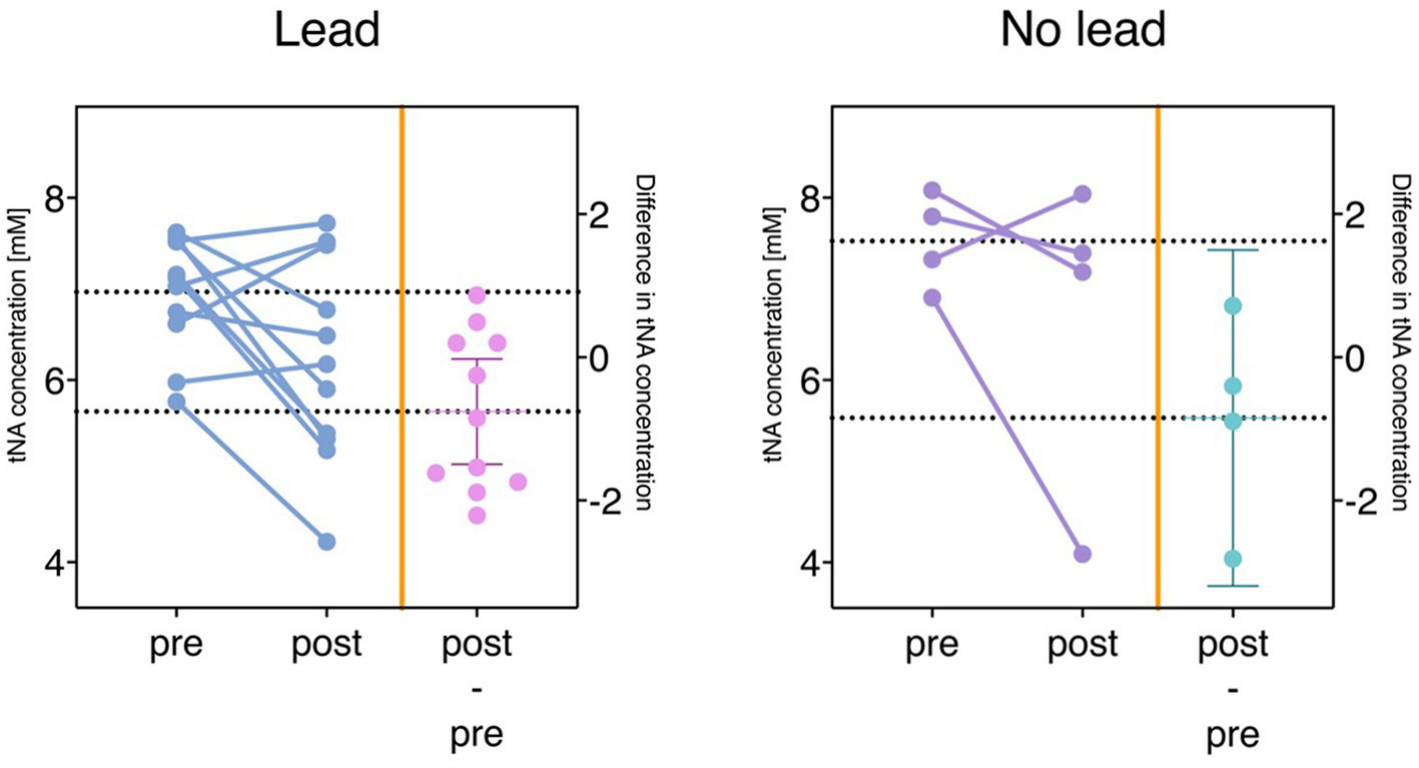
Thalamic tNA (N-acetylaspartate and N-acetyl-aspartyl-glutamate) concentration and postoperative change in tNA. The **left panel** presents data from all MRS VOIs containing a lead in the thalamus, while the **right panel** shows tNA levels in the contralateral thalamus of unilaterally operated patients. Difference in tNA concentration (postoperative – preoperative tNA) on the right side of the respective image. A significant difference in tNA was only evident in the thalamus that received a lead and not seen in the untargeted thalamus in patients with unilateral lead implantation.

Thalamic tNA concentrations differed significantly for the patient group when comparing postoperative [mean ± SD (6.39 ± 0.56)] and preoperative [mean ± SD (7.06 ± 0.38)] values (*p* = 0.006). Corresponding values in healthy controls [mean ± SD (7.35 ± 0.53)] also differed significantly from the postoperative measurements (*p* = 0.005), as shown in Table 1 and Figure 2. This significant difference in tNA was only observed in the patient's thalamus that had been targeted with a lead. In patients who received unilateral thalamic lead placement, no difference was found on the contralateral untreated side, as shown in Figure 3. No additional differences in metabolite concentrations (tCr, tCho, Glx) were observed in the thalamic voxel, and none of the studied metabolites (tNA, tCr, tCho, Glx) showed detectable differences in the cerebellar voxels (dentate nucleus and cerebellar cortex).

**Table 1:** The resulting means and standard deviations (SD) of metabolite concentrations in mM (tCr, tNA, tCho, Glx)^*^ were computed in the thalamus, dentate nucleus, and cerebellar cortex of healthy controls and ET patients preoperatively and postoperatively.

	**HC**		**ET**		**ET post op**		**HC vs. ET**	**Pre vs. post op**
**Anatomy and metabolites**	Mean (mM) (*n* = 7, 6, 7)^**^	**SD**	**Mean (mM) (*n* = 10)**	**SD**	Mean (mM) (*n* = 8, 8, 9)^***^	**SD**	***p*-value**	***p*-value**
**Thalamus**								
tCr	4.94	0.47	4.92	0.48	4.71	0.71	n.s	n.s
tNA	7.35	0.53	**7.06**	0.38	**6.39**	0.56	n.s	**0.006**
tCho	1.48	0.20	1.42	0.16	1.35	0.29	n.s	n.s
Glx	8.56	0.51	8.58	1.21	8.25	2.49	n.s	n.s
**Dentate nucleus**								
tCr	4.47	0.43	4.38	0.51	4.82	0.51	n.s	n.s
tNA	7.36	0.53	6.67	0.97	6.47	0.81	n.s	n.s
tCho	1.59	0.20	1.65	0.16	1.58	0.34	n.s	n.s
Glx	8.25	2.84	8.43	1.03	9.63	2.97	n.s	n.s
**Cerebellar cortex**								
tCr	8.32	0.63	8.01	0.83	7.94	0.76	n.s	n.s
tNA	6.30	0.40	6.60	0.37	6.19	0.76	n.s	n.s
tCho	1.60	0.29	1.74	0.23	1.63	0.22	n.s	n.s
Glx	11.05	0.62	10.61	1.20	10.08	1.74	n.s	n.s

Significant concentration differences are shown in bold.

^*^tCr, total creatine and phosphocreatine; tNA, N-acetylaspartate and N-acetyl-aspartyl-glutamate; tCho, total choline, phosphocholine, and glycero-phosphocholine; Glx, total Glu and Gln.

^**^In dentate nucleus, bilateral measurements in one HC was excluded.

^***^One patient declined surgery, and bilateral measurements were excluded postoperatively for one patient in the thalamus and for another in the dentate nucleus.

### Metabolite change in relation to ETRS

The resulting postoperative tNA values and the calculated tNA difference (postoperative – preoperative) in the thalamus implanted with a lead were not correlated with the change in ETRS scores (postoperative – preoperative).

## Discussion

We present novel metabolite information using the non-invasive technique of MRS in a highly selected patient group affected by ET, which is one of the most prevalent movement disorders (1). The most interesting findings were the observed differences in thalamic tNA concentrations following DBS, which were seen both in comparison with preoperative thalamic values and those of the healthy control group.

The group of ET patients planned for DBS surgery was particularly well-suited to study ET pathophysiology and DBS mechanism because of the severity of symptoms, and also the beneficial effect of surgery on these symptoms. Presumably, we hypothesized that this would result in significant differences, despite the relatively small study group. All the patients responded exceptionally well to the DBS treatment, with an average reduction in tremor severity score from a median value of 17 to 4. Moreover, the statistical analysis was intrinsically paired.

### N-acetylaspartate and N-acetyl-aspartyl-glutamate (tNA)

The tNA concentration in the thalamus was altered significantly after a DBS intervention when comparing tNA preoperatively and postoperatively, which demonstrated a DBS effect on the metabolite concentration in the thalamus with the lead. No corresponding difference in thalamic tNA was observed when comparing non-operated ET patients with healthy controls, which has been a consistent finding in previous MRS studies (15, 16, 19).

The most prominent spectral resonance is typically that of tNA, which consists of a combination of N-acetyl-aspartate (NAA) and the neurotransmitter N-acetyl-aspartyl-glutamate (NAAG). To distinguish between these two metabolites is difficult due to the large extent of spectral overlap. The NAA/NAAG ratio varies across the human brain, but the tNA is nevertheless nearly constant in most brain regions (20). The net decrease in tNA may therefore represent a combined decrease of both NAA and NAAG. However, the reduction of tNA could possibly also underestimate the decrease in one metabolite concentration by a concomitant increase in the other. One such example is the opposite reaction to visual stimuli, for which a NAA decrease and NAAG increase during visual stimulation have been reported (21). Since the signal is not separated in this study, the part of the tNA signal that is altered is not determined.

NAA is mainly found within neurons, dendrites, and axons (22) and it has been suggested as a biomarker for viable neurons (23). Reduced levels have been linked to brain injury including trauma and ischemia. Alzheimer's disease and other dementias are also related to decreased NAA concentrations (24).

However, given the reversible nature of DBS therapy, the postoperative reduction in tNA may reflect a modulation of thalamic activity rather than indicating a permanent lesion or neurodegenerative progression. A postmortem study on DBS patients has not indicated that lead causes permanent physical damage to the adjacent brain tissue, apart from what appears to be a mild thin tissue reaction around the lead tract. In areas of lead stimulation compared to areas with no active contact, there was no difference in tissue reaction (25).

NAA is involved in myelinogenesis and can also act as an osmolyte, and it may also be a biomarker for mitochondrial metabolism. Moreover, NAA is also a precursor of the neurotransmitter NAAG, which can regulate other neurotransmitters including modulating glutamatergic transmission (24). Reversible changes in the ratio between tNA and tCr have been demonstrated using MRS in MS lesions (26), and a decrease in tNA can represent an impairment of the mitochondrial respiratory chain without any cell loss (27).

The ratio tNA/tCr is normalized in the ipsilateral and the contralateral hemispheres following successful resection of the epileptic focus (28). There is also evidence of a dynamic tNA MRS signal associated with visual stimulation in the visual cortex by a decrease of tNA during stimulation: a change that recovers to baseline after cessation of the visual stimulus (29). The DBS lead occupies a certain volume in the thalamus voxel postoperatively, but by using water-scaled concentrations, the potential influence of the lead volume on the metabolite concentrations was compensated for because the water would be equally affected by the lead volume in the voxel. Furthermore, lead did not appear to affect the quality of the MRS measurement postoperatively. One may therefore speculate that the altered neuronal firing induced by the DBS (6) may lead to a changed metabolic activity locally in the thalamus, as is shown by the reduction of tNA.

The reduction in tNA may thus reflect either microstructural damage caused by the lead, or alternatively, neuromodulatory effects. To better delineate which of these mechanisms accounts for the observed tNA reduction, future studies should include an additional group with delayed initiation of stimulation. This group would undergo MRS assessment prior to activation of the DBS system, after sufficient time has passed for 'peri-electrode edema' to resolve, and in the absence of a microlesioning effect. Such an approach may help clarify whether the reduction in tNA is primarily attributable to the presence of a lead or to neuromodulatory processes. The finding of thalamic tNA reduction following DBS surgery requires further investigation to conclude its underlying cause.

### Other metabolites

No additional changes in metabolite concentrations and/or asymmetry were observed in the voxels placed in either the thalamus, the dentate nucleus, or the cerebellar cortex. In addition to the lead not appearing to affect spectral quality, any potential magnetic distortion it may have caused would be expected to influence all of the metabolites investigated. Since tNA was the only metabolite that was affected, this supports the interpretation that the electrode itself did not influence the results.

The Glx concentration measured in MRS is less certain than the other metabolites due to the complex spectra with multiple sub-resonances. Glu (the main component of the Glx resonances) is an excitatory neurotransmitter, and Gln is the main precursor for neuronal Glu; together, they constitute the Glx level (30). Interestingly, DBS therapy has been proposed to normalize cerebellar overactivity (31), and the dentate nucleus gives the major outflow from the cerebellum and projects to Vim, which is targeted by DBS (4). However, we did not observe a significant difference in Glx between pre-op and post-op.

Previous studies have shown a lower ratio of tNA/tCr in the cerebellar cortex in ET patients (13, 14). Another study found an increased level of Glx in the thalamus in ET patients (16). These findings represent heterogeneous results in metabolite composition, and there are several reasons why direct comparison among these studies and this study is difficult.

The classification of illness severity is not uniform, and there are differences in illness severity in the cohorts. Previous studies have primarily focused on healthy patients, and the evaluated brain regions are not entirely comparable due to variations in voxel placement and size, resulting in different degrees of partial volume effects. Moreover, acquisition parameters, field strength used, and different kinds of metabolite ratios are also diverse in the literature (13–16, 19).

### Tremor severity

ETRS difference was not correlated with the reduction of tNA in the thalamus with lead in place, when the stimulation was turned off. Clearer distinctions in the concentration of tNA would probably have resulted if the system had been ON. However, such measurements were not possible to achieve due to the restrictive MR conditions in the DBS systems used. The measured difference in tNA between pre- and postoperatively conditions may, however, point out the remains of a clinically significant DBS mechanism.

### Limitations and strengths

This study has some limitations. We may, for example, have been able to observe additional differences in metabolite concentrations if there had been no recovery period during the OFF-stimulation period. Thus, the changes in the metabolite array, which are likely associated with the tremor reduction, may have been missed. The determined difference in tNA concentration was, in our view, not sufficiently large to cause efficient tremor reduction since the symptoms returned almost immediately after the system was turned off.

Small differences in voxel placement likely exist because of anatomical variations such as ventricle size and shape, cerebellum size, and cerebellar cortex atrophy. Thus, an exactly comparable placement of the voxels among the patients was difficult because of individual anatomical differences. Partial volume effects are also expected, as the relatively large size of the MRS voxels likely resulted in the inclusion of surrounding tissues, such as adjacent white matter near the cerebellar cortex or the dentate nucleus, in addition to the intended target structure. Simultaneous examination of the unaffected brain included in the voxel may have obscured the measurement of relevant metabolic differences after the introduction of DBS therapy. Furthermore, we only measured a relatively limited volume of the cerebellar cortex. Subtle spectroscopic differences may not have been detected due to the limited size of the patient cohort. Investigation of the same-subject repeat measurement variability was not performed. However, given the statistical methods we have used, we do not believe that variability explains the observed differences in the measurement of tNA.

A key strength of the study is the inclusion of only patients who are severely affected by tremor and who demonstrate a strong response to treatment. However, this also resulted in a highly homogeneous cohort, which may have limited the ability to detect correlations with ETRS scores. Furthermore, a correlation between ETRS and a significantly changed metabolite concentration may have been observed if more patients had been studied, especially by including ET patients who are less affected by ET. An additional limitation is the lack of diversity in the study population, as all participants were Caucasian.

### Future

Future technological advances with more focused voxel sizes that can delineate a region of interest and thus, specifically examine a smaller fraction of the affected anatomy, and to evaluate metabolite concentrations when the system is ON, would be of particular interest. Moreover, MRS spectra are derived from a combination of molecular moieties whose biochemistry is complex and interconnected. For example, MRS methods that can separate NAA from NAAG resonances would be able to reveal more details of the induced alterations. In addition, exploring the long-term effects after DBS would be warranted. Moreover, to investigate whether the observed reduction in thalamic tNA concentrations is related to a more generalized neurodegenerative process, a potential continuation of this study could include thalamic volumetric analyses.

## Conclusion

We report a novel and potentially clinically relevant finding of reduced thalamic tNA concentration following DBS implantation. No other concentration changes in metabolites were observed, suggesting a localized, tNA-specific treatment effect. By increasing the understanding of which metabolic systems are potentially deranged in ET and altered after DBS, the etiology of ET can be clarified, and the mechanisms of DBS will be further understood.

## Data Availability

The data that support the findings of this study are not publicly available due to patient confidentiality. Requests to access the datasets should be directed to the corresponding author.
